# Models to estimate biological variation components and interpretation of serial results: strengths and limitations

**DOI:** 10.1515/almed-2020-0063

**Published:** 2020-08-10

**Authors:** Jorge Díaz-Garzón Marco, Pilar Fernández-Calle, Carmen Ricós

**Affiliations:** Comisión de Calidad Analítica, SEQC^ML^ , Barcelona, Spain; Servicio Análisis Clínicos, Hospital Universitario La Paz, Madrid, Spain

**Keywords:** biological variation, methods, statistical design

## Abstract

Biological variation (BV) has multiple applications in a variety of fields of clinical laboratory. The use of BV in statistical modeling is twofold. On the one hand, some models are used for the generation of BV estimates (within- and between-subject variability). Other models are built based on BV in combination with other factors to establish ranges of normality that will help the clinician interpret serial results for the same subject. There are two types of statistical models for the calculation of BV estimates: A. Direct methods, prospective studies designed to calculate BV estimates; i. Classic model: developed by Harris and Fraser, revised by the Working Group on Biological Variation of the European Federation of Laboratory Medicine. ii. Mixed-effect models. iii. Bayesian model. B. Indirect methods, retrospective studies to derive BV estimates from large databases of results. Big data. Understanding the characteristics of these models is crucial as they determine their applicability in different settings and populations. Models for defining ranges that help in the interpretation of individual serial results include: A. Reference change value and B. Bayesian data network. In summary, this review provides an overview of the models used to define BV components and others for the follow-up of patients. These models should be exploited in the future to personalize and improve the information provided by the clinical laboratory and get the best of the resources available.

## Introduction

The concept of biological variation (BV) was first formulated by Harris and Fraser in the mid-twentieth century [1]. Since then, multiple prospective experimental studies of different levels of complexity have been conducted to produce accurate BV estimates that could serve as reference values and be generalized to a diversity of populations, settings and conditions.

BV has two components: within-subject and between-subject variation. Within-subject variation is defined as the fluctuation of a measurand around its homeostatic setting point within the same subject, whereas between-subject BV is described as the variation between the homeostatic points of different subjects [1], [2], [3]. Both estimates are expressed as coefficients of variation and are referred to as CV_I_ and CV_G_, respectively, in accordance with the standard terms recommended by Simundic et al. [4].

BV has a multiplicity of applications in the clinical laboratory, including internal control of the analytical process, external quality assurance programs, estimation of the reference change value (RCV) [1], verification and validation of analytical methods and as the criterion for establishing the maximum allowable deviation caused by biological sample instability [5]. BV is also used in interference studies and for establishing the limit of quantification of a measurand [6].

There are two ways to link the concept of BV to statistical modeling. On the one hand, some models are used for generation of BV estimates (within- and between-subject variation). On the other hand, other models are built based on BV in combination with other factors in order to establish intervals that help clinicians to interpret serial results in an individual.

## Models for the generation of BV estimates

There are two types of statistical models for the calculation of BV estimates:

- Direct methods: Prospective studies specifically designed to calculate BV estimates
  - classic model: developed by Harris and Fraser, revised by the Working Group on Biological Variation of the *European Federation of Laboratory Medicine* (EFLM) (EFLM-BVWG)
  - mixed-effect models
  - Bayesian model
- Indirect methods: Retrospective studies to derive BV estimates from large databases–*Big data.*

Understanding their characteristics of these models is crucial as they determine their applicability in different settings and populations.

### A. Direct methods

#### i. Classic method

The classic method, developed by Fraser and Harris in the 60s, is still used in most studies aimed at the generation of BV estimates, fitting the model more or less strictly. The Analytical Quality Commission of Spanish Society of Laboratory Medicine (SEQC^ML^) published in 1997 a database that compiled all studies published to date on BV, that was periodically upgraded until 2014 [7], [8]. This database has been a valuable tool for laboratory professionals.

The classic model described by Fraser and Harris, which requires that samples are assayed in duplicate, assumes three requirements: BV data must be normally distributed; the distribution of variables must be homoscedastic (homogeneity of variance across the whole interval of concentrations, which implies that the distribution of results is homogeneous between the replicates of the samples and between the samples of different subjects) and finally, subjects cannot show any trend throughout the study period (stable status). The statistical method recommended for the calculation of BV estimates consists of a nested analysis of variance (ANOVA) after a thorough search for outliers at three levels (between duplicates, within subjects and between subjects)

This approach is based on the concept that the total variation of all measurements, expressed as coefficient of variation (CV_T_) is the sum of preanalytical (CV_PRE_), analytical (CV_A_) and within-subject BV (CV_I_). Once analytical variation is known and controlled, and in standard preanalytical conditions, this last component can be ruled out, thereby having only CV_I_ left in the equation. CV_I_ must be equal to the difference between total variation and analytical variation.

$$\text{VT}={\text{CV}}_{\text{PRE}}+{\text{CV}}_{\text{A}}+{\text{CV}}_{\text{I}}\to {\text{CV}}_{\text{I}}={\text{CV}}_{\text{T}}-{\text{CV}}_{\text{A}}$$

However, upon this model, for BV estimates to be robust, a set of requirements must be met. The number of subjects, samples per subjects and replicates per sample must be adequate. To such purpose, the ratio of analytical imprecision of the measurement method to the expected CV_I_ must be considered [9]. It is recommended that such a ratio should be <1, and it would be desirable to be <0.5 [1].

The EFLM-BVWG, in cooperation with the *Task Group on Biological Variation Databa*se, recently published a review of studies using a BV data critical appraisal checklist designed by the EFLM-BVWG itself. Thus, studies were classified into four categories (A, B, C and D) in ascending order of quality. A meta-analysis was carried out to estimate the components of BV, CV_I_, and CV_G_ and their 95% confidence intervals [10]. The new BV database was published on the EFLM website [11] in May 2019. Most of the studies published and included in the new database are adapted to the classic model. However, in most of these studies, CV_I_ estimates were obtained by simple subtraction of variances (subtracting the analytical variation from the total observed variation) instead of using a most appropriate statistical method such as ANOVA. As a result, these studies have a poorer methodological quality. Other limitations of these studies are related to the assumption of individual stability, the method used to calculate CV_A_, the homoscedasticity of variances and the method for testing outliers [10], [12].

The most relevant advantage of this approach is that it has been extensively tested and validated, and it is widely used and known. It is a prospective method wherein preanalytical variables are well controlled, which guarantees the robustness of estimates.

However, this method has some limitations:

Some measurands may not meet homoscedasticity requirements.
If the process of testing outliers is carried out with excessive zeal, so that even moderate outliers are eliminated, the transferability and statistical power of the study are limited. This is due to the manual processing of outliers even though some standard criteria are fulfilled (Reed, Cochrane, etc.). Valuable results that are representative of the subjects may be eliminated.
Most studies use a small sample of subjects to reduce costs and facilitate the study. In addition, the number of samples obtained per subject (visits) is not generally high as it compromises the participant’s adherence to the study.
The presence of significant trends that affect the stability of the subject (homeostasis) over the study period may compromise the generation of reliable estimates. If trends are observed in a certain subject, this subject could be considered as an outlier. If instead trends affect all results such as in the case of seasonal variations, corrections can be performed to minimize the effects of this factor. Unfortunately, no recommendations or clear criteria have been established in the literature to solve this problem.
The possible presence of noncontrolled variables, as observed by the researcher, may cause bias in the obtained estimates.

#### ii. Mixed-effects models

The formula used in this method is the same as the one of the classic models as it is based on a nested ANOVA. However, the estimation is performed using a mixed model that includes further variables apart from homeostatic regulation. In this model, it is also necessary that results are normally distributed, and homogeneity of variance should be checked for BV estimates to be reliable [13]. The main difference with respect to the classic method is that it may include other variables that may affect the BV of the studied measurands. These variables might be included in the model, and the magnitude of their effect on the BV is measured (influence).

In general, this model includes two types of effects: fixed effects, which are variables with potential influence (age, sex, medication, health status, to name a few), and random effects, which are BV estimates and imprecision (CV_I,_ CV_G_ and CV_A_).

The main advantage of this model relies on the ability to determine if BV is affected by any variable and to measure such an effect. On the other hand, if the variables potentially affecting BV are previously identified, outlier handling can be less rigorous [13].

This model has the same limitations as the classic model but adds the difficulty of requiring previous awareness of potentially influencing variables. In addition, this statistical method is more complex and requires advanced mathematical skills. For several variables to be included simultaneously in this model, a very large sample size is required. Finally, an important limitation of this model is that it has not been fully validated in BV studies [13].

#### iii. Bayesian model

This model was recently developed and published by Røraas et al. [14] that is based on a Bayesian method that does not require the variances to be homogeneous. Thus, this model loosens strict outlier handling, thereby preventing that potentially valuable data for BV estimation are lost [9].

The fact that this model does not require a three-level outlier analysis and homoscedasticity simplifies and automates BV component estimation, which makes these estimates more reliable and, even more important, standardizable. In addition, this method can be used by researchers to estimate individualized CV_I_, allowing to have the estimates expressed as percentiles (median and interquartile range for CV_I_ and CV_G_), instead of yielding a central value as other models do (mean).

This model has been validated against traditional methods and has demonstrated to yield similar BV estimates. To perform these comparisons, Røraas et al. [14] used the data obtained in the EuBIVAS study for chloride and triglycerides [15]. In other words, this model has already been validated with real BV data from robust studies, which guarantees the validity of this approach.

However, the main limitation of this model is the level of mathematic and programming skills required to be able to operate the statistical software needed for its implementation. Another disadvantage is that this model requires the use of BV estimates from previous studies as *a priori* information (hyperparameters); therefore, if the *a priori* information entered in the model is not correct, the conclusions obtained may be incoherent or inconsistent with the hypotheses defined in the design of the study.

In summary, this type of model should only be used when solid statistical support is available. This model is extremely useful to study BV in nonhomoscedastic measurands that yield a high percentage of outliers that cannot be explained by the information recorded during the study.

### B. Indirect methods – Big data

Although this method has gained popularity in recent years, few studies have been conducted, and consensus has not been reached on its optimal design (statistical method, inclusion criteria, among other factors).

This strategy is based on the assumption that most estimates are not affected by health status because abnormal results are excluded after an outlier analysis [16]. Some examples include the studies conducted by Loh et al. [17] and Loh and Metz [18] in the pediatric population assisted in primary care, with similar CV_I_ results to those obtained in healthy adults.

The most relevant advantages of this method are that it enables assessing differences by age, gender, study duration [16], [17], [18] and even between different diseases [19]. As the number of subjects included in this type of studies is larger than that in the prospective studies (up to several thousand because the method is based on the laboratory database), they have a higher degree of generalizability and statistical power. This method is economical as it requires limited human and material resources and does not require an experimental phase.

Some of its limitations are that preanalytical variables are not standardized; the homeostatic status of the subjects is unknown; CV_A_ is derived from the quality assurance results (stabilized control samples with a different matrix from patients’ samples and different concentrations from those in patients’ samples) instead of duplicate analysis measured in subjects’ samples. These studies often have a low number of samples per subject and variable sampling intervals, which, in the case of some measurands, may limit the robustness of results. Some of these multicentric studies include results from different measurement methods and reagent lots.

## Models for defining ranges that help in the interpretation of individual serial results

The following are the models for defining ranges that help in the interpretation of individual serial results:

- Reference change value
- Bayesian data network

### A. Reference change value

The RCV defines an interval from which the difference between serial results from an individual could be considered biologically significant. The RCV is derived from the measurement of an analytical error and the fluctuation of a measurand within an individual. For such purpose, the laboratory’s CV_A_ and the CV_I_ are included in the calculation [20]. This concept is based on the assumption that all individuals have the same CV_I_, and it requires robust and representative CV_I_ from the population to which this concept is applied for monitoring. The RCV is calculated using the following formula, with a Z probability of 1.96 and 1.65 for bidirectional and unidirectional changes, respectively [1]:

$$\text{ RCV}=2^{1/2}\text{Z}{\left({\text{CV}}_{\text{A}}^{2}+{\text{CV}}_{\text{I}}^{2}\right)}^{1/2}$$

In contrast with the classic model, which is based on the assumption that all measurands are normally distributed and the interval is symmetrical, the method currently recommended for the calculation of the RCV is based on a logarithmic method that defines an asymmetrical interval [21]. The formula is as follows:

$${\text{RCV}}_{\text{pos}}=\left(\exp\left(1.96\cdot 2^{1/2}\cdot \alpha \right)-1\right)\cdot 100$$

$${\text{RCV}}_{\text{neg}}=-\left(\exp\left(1.96\cdot 2^{1/2}\cdot \alpha \right)-1\right)\cdot 100$$

$$\alpha ={\left(\text{Ln }\left({\text{CV}}_{\text{T}}+1\right)\right)}^{1\text{/}2}\text{;}\;{\text{CV}}_{\text{T}}={\text{CV}}_{\text{A}}+{\text{CV}}_{\text{I}}$$

The measurands that can be interpreted by this method are those that are subject to a greater homeostatic regulation, i.e., measurands with a low index of individuality (II < 0.6) as in the case of creatinine, electrolites and some hematology parameters. This index relates within- and between-subject BV estimates (II = CV_I_/CV_G_) [1]. The serial results of measurands with a high individuality (low II) should be interpreted by the RCV as an alternative to biological reference intervals.

The main limitation of this concept is that it is in unknown to most clinicians, and laboratory information management systems are not fully adapted to this model.

Another aspect to be considered is that preanalytical conditions have been standardized in most of the BV studies to avoid the influence of preanalytical factors and bias when calculating BV estimates. However, in a real setting, these variables are not always standardized by laboratories, and this source of variation is not considered when the range is defined. In this context, RCV could be too strict, and changes between serial results could be interpreted as biologically significant when they are not.

In addition, CV_A_ included in the equation is derived from the internal quality control. This implies that in measurands whose CV_A_ depends on the measurand concentration, a different CV_A_ should be applied for each concentration, based on the results obtained. This limitation hinders the application of the model.

### B. Bayesian data network

Bayesian models make it possible to update and adjust the probability of obtaining a result within a range based on previous information. These models are flexible and improve progressively as further information is integrated. At baseline, previous information can be obtained from the literature or reliable sources [11].

These models have been developed by Sottas et al. [22] in the field of laboratory medicine and are applied by the World Anti-Doping Agency in the detection of illicit drug consumption. The method used in the follow-up of athletes is named biological passport. Instead of detecting the illicit substance in the body, indirect markers of doping are monitored (hemoglobin and reticulocytes, among others).

To predict the specific interval of variation of a measurand in a subject, the model incorporates previous information based on both result distribution in the specific population group to which the subject belongs (age, gender, ethnicity, disease) and individual results [23].

This model relies on a hierarchical Bayesian network, consisting of several levels, that is built from patient results and heterogeneity variables such as gender, age and other factors. In the first level, it separates the data into different distributions (groups) as a function of these heterogeneity variables. In the second level, this model uses BV estimates derived from these groups as *a priori* information (hyperparameters) [24].

Then, after including the subject results in the model, distributions that define a range predicting the individual result with a higher degree of robustness than in former levels are obtained.

In sum, this model defines specific reference intervals for a subject based on previous information. In other words, it is based on preliminary distributions and incorporates data that provide information to the model, which generates more robust distributions. In addition, if the volume of previous results included in the database for the population of study is high enough, previous specific BV studies would be not necessary (hyperparameters).

According to the literature, the number of patients or samples needed to meet the requirements of a clinical trial may decrease significantly, or the trial may be completed earlier if this model is used. For instance, Sottas et al [24] indicate that when population-based reference intervals are used, 600 subjects are needed to detect a change of 0.06 mg/dL in serum creatinine concentrations. In contrast, if an analysis of covariance with respect to basal values is performed, 210 subjects are needed, whereas 20 subjects are required if the Bayesian model is used. The use of this model in clinical practice may help clinicians in the interpretation of laboratory results [24].

The Bayesian network model assumes that there is no analytical error. To achieve that, all results must be obtained using the same analytical procedure, which guarantees the absence of bias and ensures low and fully controlled imprecision. However, many of the measurands routinely assayed in clinical laboratories for disease monitoring are not measured under these conditions. Indicators of analytical error obtained by internal quality assurance strategies could be incorporated into this model as hyperparameters to obtain a more robust prediction of ranges derived.

Another limitation is that these databases are not public, and they cannot be freely accessed by clinical laboratories.

In summary, this review aimed to give an overview of the models used to define BV components as well as others for patient monitoring. It should be exploited in the future to personalize and improve the information provided by the clinical laboratory, taking advantage from the available resources.
